# An architectural switch in the evolution of the γ-tubulin ring complex

**DOI:** 10.64898/2026.08.03.742184

**Published:** 2026-08-04

**Authors:** Rościsław Krutyhołowa, Yi Xie, Banyon Carnell, Hugo Muñoz-Hernández, Daniel Zhang, Florina Marxer, Shaul Yogev, Michal Wieczorek

## Abstract

Canonical microtubules contain 13-protofilaments and are templated by the γ-tubulin ring complex (γ-TuRC). However, some eukaryotes assemble non-canonical microtubules, like the 11-protofilament structures found in *Caenorhabditis elegans* . How γ-TuRCs adapt to template alternative microtubule geometries is unclear. Here, we present the cryo-electron microscopy structure of the *C. elegans* γ-TuRC (γ-TuRC *_Ce_* ), revealing a cone-shaped assembly consistent with an 11-protofilament template. While the complex incorporates the conserved subunits actin, GCP2 and GCP3, γ-TuRC *_Ce_* replaces GCP4-6 with a divergent 4-spoked assembly containing additional copies of GCP2 and the nematode-specific proteins GTAP-1 and GTAP-2. Structures of nucleotide-free γ-TuRC *_Ce_* subcomplexes reveal partial γ-tubulin unfolding, suggesting nucleotide binding stabilizes eukaryotic tubulins. Remarkably, reconstituted 4-spoked assemblies can multimerize into ∼13-fold symmetric microtubule nucleation templates *in vitro* , contrasting with the native complex’s 11-protofilament architecture. Our work defines the structural blueprint of an 11-protofilament microtubule template and shows how divergent γ-tubulin components are repurposed to accommodate non-canonical microtubule lattices.

## Full Text Availability

The license terms selected by the author(s) for this preprint version do not permit archiving in PMC. The full text is available from the preprint server.

